# Tibiofemoral Subluxation on Radiograph as a Predictor of Location and Size of Osteochondritis Dissecans Lesions of the Knee

**DOI:** 10.1177/23259671241232397

**Published:** 2024-03-06

**Authors:** Marco-Christopher Rupp, Felix Hochberger, Daniel P. Berthold, Lukas N. Muench, Andreas B. Imhoff, Sebastian Siebenlist, Lukas Willinger

**Affiliations:** * Department of Sports Orthopaedics, Technical University Munich, Munich, Germany; † Department of Orthopaedics and Trauma Surgery, Musculoskeletal University Center Munich (MUM), University Hospital, Ludwig Maximilian University of Munich (LMU Munich), Munich, Germany; Investigation performed at the Department of Sports Orthopaedics, Technical University Munich, Munich, Germany

**Keywords:** impingement, knee joint line obliquity, lower leg alignment, osteochondritis dissecans, subluxation, valgus, varus predisposition

## Abstract

**Background:**

Lower limb malalignment has been associated with osteochondritis dissecans (OCD). However, the location of the OCD lesion often is not concordant with the mechanical leg axis. Other potentially modifiable alignment parameters may influence the propensity for impingement of the femoral condyles.

**Purpose:**

To assess differences in lower limb alignment (LLA) and relative tibiofemoral position between patients with medial (MFC-OCD) or lateral OCD (LFC-OCD) of the femoral condyle.

**Study Design:**

Cohort study; Level of evidence, 3.

**Methods:**

Patients ≤30 years old who were diagnosed with unicondylar OCD between January 2010 and January 2020 were eligible for this study. Included were 55 patients (age, 20.8 ± 4.5 years)—46 with MFC-OCD and 9 with LFC-OCD. Preoperative standing long-leg radiographs were studied to obtain primary outcomes—including LLA and mechanical alignment analyses—and secondary outcomes—including knee joint obliquity angle; rotation angle; medial, central (c-subluxation), and lateral subluxation (L-subluxation) of the tibia relative to the femur in the coronal plane; and tibiofemoral joint line center distance (TFJCD).

**Results:**

With regard to primary outcomes, LLA was significantly different between MFC-OCD (1.7°± 3.1° varus) and LFC-OCD (2.7 ± 3.1° valgus) (*P* < .001), and 78% (36/46) of patients with MFC-OCD had varus alignment, whereas 78% (7/9) of patients with LFC-OCD had valgus alignment (*P* < 0.002). With regard to secondary outcomes, patients with MFC-OCD had a more medial tibial position in relation to the femur, with a significantly smaller rotation angle (5.6°± 2.4° vs 9.6°± 3.6°; *P* < .001), a smaller C-subluxation (7.2 ± 6.6 vs 14.9 ± 8.8 mm; *P* < .01), a smaller L-subluxation (2.3 ± 2.6 vs 4.4 ± 2.7 mm; *P* < .05), and reduced TFJCD (3.5 ± 1.7 vs 6.6 ± 1.8 mm; *P* < .001) compared with the LFC-OCD group. For patients with MFC-OCD, the size of the OCD was significantly correlated with C-subluxation (*r* = 0.412; *P* = .006).

**Conclusion:**

LLA was significantly different according to OCD location. In patients with MFC-OCD, the tibia was subluxated medially, resulting in a change of joint geometry by approximation of the medial tibial eminence toward the medial femoral condyle, potentially causing excessive pressure overload and microtrauma of the cartilage. Interestingly, the extent of subluxation was correlated with OCD size.

Osteochondritis dissecans (OCD) is an osteochondral pathology affecting the subchondral bone with or without the overlying cartilage.^3,21^ It predominantly affects male patients,^21^ with a peak of incidence at 10 to 20 years of age.^24^ While the exact etiopathogenesis remains unclear, OCD was shown to be associated with several biological ^4,13,28,34^ or mechanical factors—such as lower limb malalignment,^7,15,18^ anatomic abnormalities,^9,11,20^ and repetitive microtrauma in overuse situations.^4,17^ These factors are suggested to act synergistically, resulting in ischemia of the subchondral bone^4^ and subsequent insufficiency fracture.^4,31^

Within the spectrum of anatomic and mechanical factors, previous studies have suggested a role of discoid menisci,^27^ external tibial torsion,^6^ femoral condyle prominence,^20^ coronal and anterior-posterior tibial slope^33^ as well as malalignment of the mechanical axis^7,15,18^ in the development of an OCD. More specifically, varus or valgus malalignment has been shown to be associated with OCD lesions in the overloaded compartment.^7,15,18^ Furthermore, the theory of tibial spine impingement suggests that impingement between the anterior tibial spine and the lateral facet of the medial femoral condyle (MFC) would result in increased shear forces and repeated microtrauma at the MFC, the most common location for OCD.^4,9^ In support of this theory, imaging studies demonstrated that patients affected by OCD had a more prominent tibial spine^9^ and decreased femoral notch width^11^ compared with unaffected patients.

Although abnormalities in mechanical leg alignment have been proposed to increase the risk of tibial spine impingement, data investigating the role of the mechanical axis^7,15,18^ and the tibial slope in the development of OCD remain sparse.^33^ However, in up to 30% to 69% of cases, the location of the OCD lesion is not concordant with the mechanical leg axis.^7,15^ Modifiable alignment parameters that may substantially increase the propensity for impingement of the femoral condyles— such as a coronal subluxation of the tibia relative to the femur^1^ or tibial knee joint line obliquity (KJLO)^32^—have not yet been investigated regarding their potential role in the development of OCD, indicating a gap in the literature. These parameters are controversial and may have clinical significance. This study aimed to address this gap in the literature by assessing differences in lower limb alignment (LLA) and relative tibiofemoral position between patients with OCD of the medial-lateral condyle (MFC-OCD) or lateral femoral condyle (LFC-OCD). It was hypothesized that there would be a significant difference in knee joint line obliquity (KJLO) and tibiofemoral position between patients with MFC-OCD and LFC-OCD.

## Methods

The study protocol of this retrospective cohort study received institutional review board approval. A review of the institutional data bank was performed to identify patients who were diagnosed between January 2010 and January 2020 with an isolated, symptomatic OCD not related to trauma and without concomitant injuries to the menisci or ligaments. In accordance with the definition of OCD,^8,25^ only patients <30 years were included in order to mitigate the influence of secondary degenerative changes due to leg geometry. Patients were excluded if they (1) had OCD lesions in multiple locations within the knee, (2) had OCD lesions in another location than the femoral condyles, and (3) did not have a long leg radiograph available for analysis. A chart review was conducted to extract the following information from the patients’ clinical and imaging records: age at first presentation; sex; height; weight; body mass index; laterality; affected; and size of the defect, as detailed in the operative and magnetic resonance imaging reports.

### Radiograph Acquisition

For the acquisition of long-leg radiographs, a consistent standardized protocol was utilized to ensure accurate and consistent positioning, full knee extension, and weightbearing on both legs. The patella was aligned anteriorly and centrally to ensure correct lower limb rotation. Depending on the patient’s height, 2 or 3 preoperative weightbearing anteroposterior radiographs were acquired. The overlapping radiographs were merged to obtain a full-standing long-leg radiograph. A ruler and reference sphere served as length reference.

### Radiographic Analysis

Measurement on anterior-posterior long leg radiographs was performed using the state-of-the-art US Food and Drug Administration–approved mediCAD orthopaedic planning software (mediCAD Classic, Knee 2D, Version 6.0; Hectec). All images were calibrated to a reference sphere to alleviate measurement errors for absolute measurement parameters. A standard analysis of alignment was defined as primary outcomes. This analysis included the mechanical femorotibial angle (mFA-mTA), mechanical lateral proximal femur angle (mLPFA), mechanical lateral distal femur angle (mLDFA), joint line convergence angle (JLCA), mechanical medial proximal tibia angle (mMPTA), mechanical lateral distal talus angle (mLDTA), and anatomic mechanical femur angle (AMA).^29^

**Figure 1.**
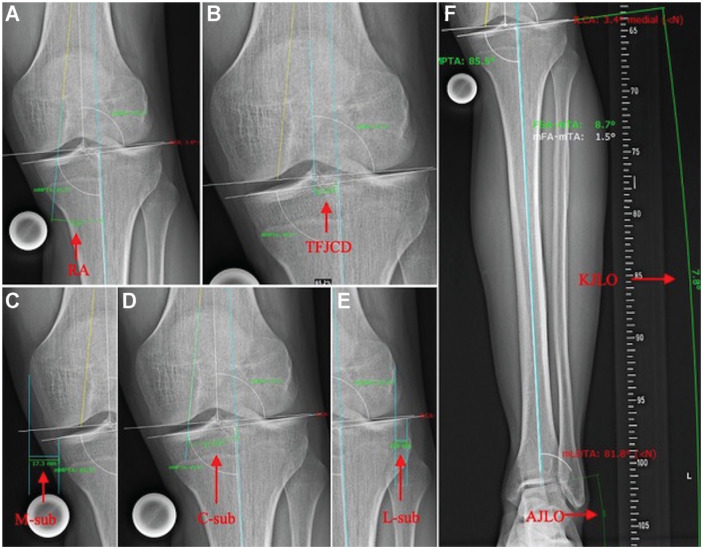
Radiographic measurements of lower limb alignment: (A) RA, the minor angle between the distal femoral and proximal tibial axes; (B) TFJCD, the distance between the center of the tibial JL and the center of the femoral JL on the tibial JL; (C) M-sub, the distance between a strictly vertical line through the most medial point of the medial femoral condyle and a vertical line through the most medial point of the medial tibial plateau; (D) C-sub, the distance between the intersection of the femoral anatomic axis and the tibial anatomic axis at the tibial JL; (E) L-sub, the distance between a strictly vertical line through the most lateral point of the lateral femoral condyle and a vertical line through the most lateral point of the lateral tibial plateau; and (F) KJLO, the angle between the tibial JL and a horizontal line parallel to the floor; and AJLO, the angle between the talar JL and a horizontal line parallel to the floor. AJLO, ankle joint line obliquity; C-sub, central subluxation; JL, joint line; KJLO, knee joint line obliquity; L-sub, lateral subluxation; M-sub, medial subluxation; RA, rotation angle; TFJCD, tibiofemoral joint line center distance.

In addition, the following measurements were performed, which were defined as secondary outcomes: KJLO, ankle joint line obliquity (AJLO), central subluxation (C-subluxation), lateral subluxation (L-subluxation), medial subluxation (M-subluxation), rotation angle, and tibiofemoral joint line center distance (TFJCD). According to previous definitions, the KJLO was defined as the angle between the tibial joint line and a horizontal line parallel to the floor,^22^ with negative values indicating medial inclination and positive values indicating lateral inclination. The AJLO was defined as the angle between the talar joint line and a horizontal line parallel to the floor,^22^ with negative values indicating medial inclination and positive values indicating lateral inclination. C-subluxation was defined as the distance between the intersection of the femoral anatomic axis and the tibial anatomic axis with the tibial plateau.^1^ L-subluxation was defined as the distance between a strictly vertical line through the most lateral point of the lateral femoral condyle and a vertical line through the most lateral point of the lateral tibial plateau^1^. M-subluxation was defined as the distance between a strictly vertical line through the most medial point of the MFC and a vertical line through the most medial point of the medial tibial plateau.^1^ The rotation angle was defined as the minor angle between the distal femoral and proximal tibial axes.^1^ The TFJCD was defined as the distance between the center of the tibial joint line and the center of the femoral joint line on the tibial joint line.^29^ A detailed measurement protocol is provided in Figure 1.

To account for differences in patients’ height and constitution, C-, L-, and M-subluxation were normalized to each patient’s tibial plateau width according to the following formula: *(C/L/M) subluxation* = *[(C/L/M) distance/tibial plateau width] × mean tibial plateau width within the cohort*. Last, OCD size was retrieved from radiologic and operative reports from the patient chart. In accordance with previous research,^7^ varus alignment was defined as −1° for adults^5,12,29^ and −0.5° in the mechanical tibiofemoral angle for adolescents.^30^

All measurements were conducted by a single orthopaedic surgeon (F.H.), with 4 years of experience in conducting measurements related to LLA. Measurements were repeated after a washout period of 2 months to determine intrarater reliability. To determine interrater reliability, measurements were repeated on 20% of the dataset by a second orthopaedic surgeon (M.C.R.), with 4 years of experience in conducting measurements related to LLA.

### Statistical Analysis

Statistical analysis was performed using SPSS Version 26.0 (IBM). The significance level for all statistical tests was set at *P* < .05. Continuous variables were reported as means ± standard deviations, and categorical variables were reported as counts and percentages. The distribution of continuous variables in the study was categorized using the Kolmogorov-Smirnoff test. According to their respective distribution, continuous variables between groups were compared employing a parametric unpaired *t* test or the nonparametric Mann-Whitney *U* test. Nominal variables were compared using the chi-square or Fisher exact tests, as statistically appropriate.

To assess the intra- and interrater reliability of the radiographic measurements, the 2-way random intraclass correlation coefficient (ICC) was used. The ICC values were interpreted as follows: <0.4, poor reliability; 0.4 to 0.75, moderate reliability; and >0.75, excellent reliability.

A Pearson correlation analysis between OCD size (as a correlate of the severity of the OCD) and the radiographic parameters was performed to detect potential associations between the severity of the OCD and expressions of radiographic alignment parameters.

To control for confounders and potential relationships between radiographic variables, a multivariable logistic regression was performed to (1) identify independent variables associated with the location of the OCD lesion and (2) calculate the individual predictive power of the variables via odds ratios. The dependent variable was defined as the incidence of an MFC-OCD. Because of sample size considerations, a forward-feature selection was performed to identify variables to be included in the regression model. To provide orthopaedic surgeons with actionable threshold values of the radiographic parameters evaluated to determine which condyle may be at risk for developing an OCD, cutoff values for the individual subluxation parameters were calculated via receiver operating characteristic (ROC) analysis. The strength of the model was determined by the area under the ROC curve (AUC), in which an AUC of ≥0.7 was deemed acceptable, and an AUC of ≥0.8 was deemed excellent.^10^ Optimal thresholds were calculated using the Youden index to maximize the sensitivity and specificity of threshold values.

A post hoc sample size calculation performed with G*Power (Erdfelder, Faul, Buchner, Lang; Heinrich Heine University)^14^—based on the observed intergroup difference of 4.4° of the primary endpoint, the mFA-mTA, with a standard deviation of 3.1° and an effect size of 1.42 at *P* < .05—revealed a statistical power of β = 0.986.

## Results

**Figure 2.**
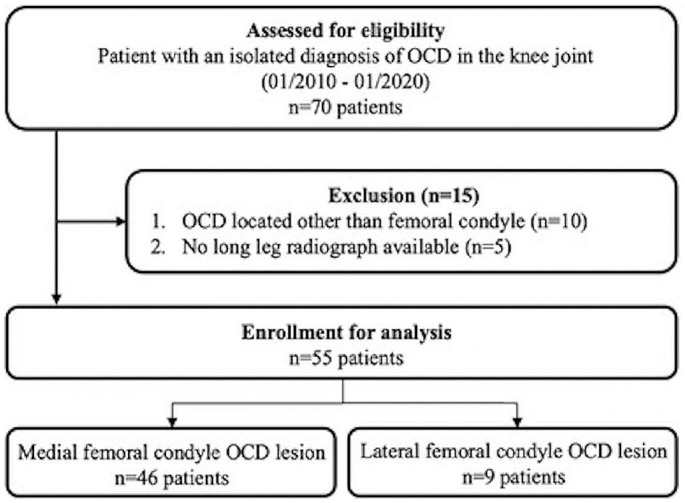
A flowchart showing the study cohort after accounting for the inclusion and exclusion criteria. OCD, osteochondritis dissecans.

Of 70 patients assessed for eligibility, 55 patients (mean age, 20.8 ± 4.5 years [42% women]) were included after the application of the inclusion and exclusion criteria; 46 patients were included in the MFC-OCD group (mean age, 21.4 ± 4.5 years; [range, 12-30 years]; 43.5% women), and 9 patients were included in the LFC-OCD group (mean age, 18.2 ± 4.5 years; [range, 14-23 years]; 33% women) (Figure 2).

### Radiological Analysis of Lower Leg Geometry

Details on the demographic and radiological variables of the MFC-OCD and LFC-OCD groups can be found in Table 1. According to the radiologic analysis of lower limb geometry, the JLCA (*P* < .001), as well as the mFA-mTA (*P* < .001), was significantly different according to the location of the OCD lesion, indicating that MFC-OCD was associated with medial convergence and lateral opening of the joint, as well as varus malalignment. The MFC-OCD group also had a significantly smaller rotation angle (*P* < .001), a smaller C-subluxation (*P* = .007), a smaller L-subluxation (*P* = 0.037), and a smaller TFJCD (*P* < .001) compared with the LFC-OCD group, indicating an L-subluxation of the femur on the tibia causing impingement on the MFC (Table 1).

**Table 1:** Comparative Analysis Between the MFC-OCD and LFC-OCD Groups^*a*^

Variable	MFC-OCD (n = 46)	LFC-OCD (n = 9)	*P*
Sex			—
Male	26 (56.5)	6 (66.7)	
Female	20 (43.5)	3 (33.3)	
OCD size, mm^2^	314.1 ± 167.1 (100 to 750)	395 ± 163.8 (200 to 600)	.562
BMI, kg/m^2^	24.5 ± 4.9 (16.8 to 39.8)	26 ± 3.5 (21.2 to 33.2)	.129
Alignment parameters			
mLPFA, deg	86.7 ± 5.9 (68.9 to 98.9)	88.8 ± 2.6 (83.9 to 92.2)	.297
mLDFA, deg	88.9 ± 2.2 (84.8 to 96.5)	87.4 ± 2.4 (83.7 to 91.5)	.07
mMPTA, deg	87.6 ± 2.5 (82.1 to 93.7)	87.9 ± 2.4 (83.1 to 90.9)	.711
mLDTA, deg	86.1 ± 4.2 (76 to 95.5)	84.7 ± 4.7 (76.2 to 90.7)	.381
JLCA, deg	0.3 ± 1.7 (–3.7 to 4.5)	−2.1 ± 1.8 (–4.9 to 0.1)	**<.001**
AMA, deg	6 ± 1.2 (2.5 to 9.1)	6.1 ± 0.6 (4.8 to 6.8)	.699
mFA-mTA, deg	−1.7 ± 3.1 (–16.4 to 5.4)	2.7 ± 3.1 (–1.7 to 7.8)	**<.001**
Rotation angle, deg	5.6 ± 2.4 (0.7 to 12.2)	9.6 ± 3.6 (4.8 to 15.7)	**<.001**
KJLO, deg	−1.6 ± 2 (–6 to 2.7)	−2.8 ± 2.2 (–6.8 to 0.8)	.106
AJLO, deg	−3 ± 5.1 (–13.1 to 9.9)	−6.1 ± 4.8 (–12.9 to 0.6)	.099
C-subluxation, mm	−7.3 ± 6.4 (–22.2 to 17.2)	−13.9 ± 6.9 (–23.2 to 2.0)	**.007**
M-subluxation, mm	−6.1 ± 4.1 (–12.8 to 6.9)	−8.1 ± 2.9 (–13.2 to −5.3)	.099
L-subluxation, mm	2.3 ± 2.7 (–6.2 to 12.4)	4.1 ± 2.4 (0.8 to 8.3)	**.037**
TFJCD, mm	3.5 ± 1.8 (0.2 to 9.5)	6.4 ± 1.7 (3.8 to 8.6)	**<.001**
KJLO^*b*^			.324
Medial inclination	31 (67.4)	8 (88.9)	
Lateral inclination	15 (32.6)	1 (11.1)	
AJLO^*b*^			.739
Medial inclination	29 (63)	8 (88.9)	
Lateral inclination	17 (37)	1 (11.1)	
Alignment			**.002**
Neutral	8 (17.4)	1 (11.1)	
Varus	29 (63)	1 (11.1)	
Valgus	9 (19.6)	7 (77.8)	

a Data are reported as mean ± SD (range) or n (%). Bold *P* values indicate statistically significant differences between groups (*P* < .05). AJLO, ankle joint line obliquity; AMA, anatomic mechanical femur angle; BMI, body mass index; C-subluxation, central subluxation; JLCA, joint line convergence angle; KJLO, knee joint line obliquity; L-subluxation, lateral subluxation; LFC-OCD, lateral femoral condyle lesion; M-subluxation, medial subluxation; mFA-mTA, mechanical femorotibial angle; MFC-OCD, medial osteochondritis dissecans lesion; mLDFA, mechanical lateral distal femur angle; mLDTA, mechanical lateral distal talus angle; mLPFA, mechanical lateral proximal femur angle; mMPTA, mechanical medial proximal tibia angle; OCD, osteochondritis dissecans; TFJCD, tibiofemoral joint line center distance.

b Nominal categorization of either lateral or medial inclination of the joint line.

### Correlation Analysis

For MFC-OCD, the size of the OCD lesion was significantly and positively correlated with the C-subluxation (*r* = 0.412; *P* = .006). None of the other radiographic parameters were associated with OCD size at either the MFC or LFC (*P* > .05).

### Multivariable Logistic Regression

Controlling for confounding variables and potential interrelations of the multivariable logistic regression, the stepwise forward-feature selection chose the mLDFA, JLCA, and the tibiofemoral joint line center point distance as independent variables for the model. The regression analysis showed that an increase of 1° in the JLCA (ie, toward a lateral opening of the joint) increased the risk for the incidence of MFC-OCD by 124% (odds ratio [OR], 2.239 [95% CI, 1.149-4.362]; *P* = .018) and that an increase of 1° in the TFJCD decreased the risk for the incidence of MFC-OCD by 55% (OR, 0.443 [95% CI, 0.219-0.896]; *P* = .024) (Table 2). The model itself showed statistical significance (*P* < .001), with a Nagelkerke *R*^2^ of 0.665 and a Hosmer-Lemeshow significance of 0.616 after the final feature selection.

**Table 2:** Results of Multivariable Regression to Identify Morphologic Factors Predictive of OCD Location^*a*^

Predictive Variable	OR (95% CI)	*P*
mLDFA	1.822 (0.976-3.402)	.06
JLCA	2.239 (1.149-4.362)	**.018**
TFJCD	0.443 (0.219-0.896)	**.024**

a Bold *P* values indicate statistical significance (*P* < .05). JLCA, joint line convergence angle, mLDFA, mechanical lateral distal femur angle, OCD, osteochondritis dissecans; OR, odds ratio; TFJCD, tibiofemoral joint line center distance.

### Threshold Calculation

**Appendix Figure A1.**
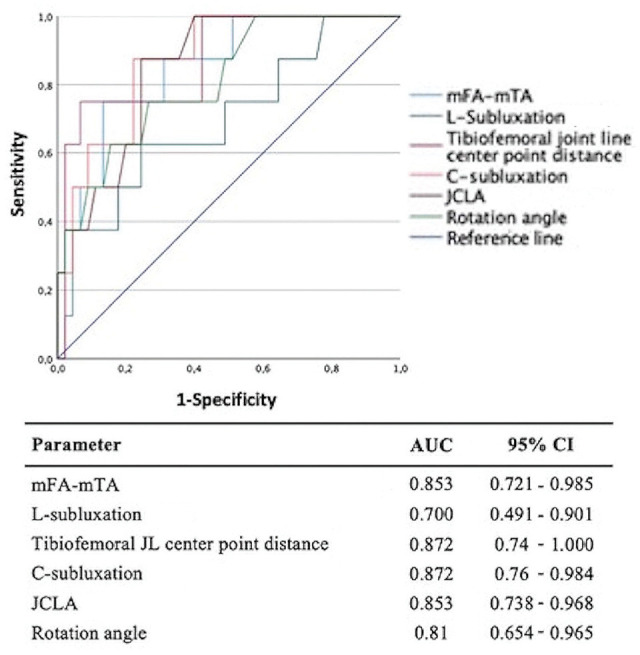
ROC curve analysis. The AUC ranged between 0.700 and 0.872, indicating acceptable to excellent performance of the model for predicting the location of OCD. AUC, area under the curve; C-subluxation, central subluxation; JLCA, joint line convergence angle; L-subluxation, lateral subluxation; M-subluxation, medial subluxation; mFA-mTA, mechanical femorotibial angle; OCD, osteochondritis dissecans; ROC, receiver operating characteristic.

In calculating an actionable threshold for the respective parameters to determine the risk for development of an OCD lesion at the MFC or LFC, the ROC analysis demonstrated acceptable to excellent predictive value of each, with AUCs ranging between 0.700 and 0.872. The cutoff values between the incidence of MFC-OCD and LFC-OCD was 1° for mFA-mTA, 3.6 mm for L-subluxation, 5.4 mm for the tibiofemoral JL center point distance, −10.12 mm for the C-subluxation, −0.4° for the JLCA, and 7.3° for the rotation angle (Appendix Figure A1).

### Measurement Reliability

The inter- and intrarater reliability values for each radiographic measurement were excellent (ICCs, 0.816-0.998), as shown in Appendix A1.

## Discussion

The main findings of this study were that LLA parameters indicative of compartment overload, such as mechanical leg axis and JLCA, were significantly different depending on the medial or lateral condylar location of the OCD lesion. Furthermore, parameters indicating a subluxation of the tibia relative to the femur were significantly different according to the condylar location of the OCD, while the extent of this subluxation was also positively correlated with the size of MFC-OCD lesions. These results suggest that specific tibiofemoral alignment parameters predisposing for tibial spine impingement may contribute to the development of OCD. In addition, this study provides a differentiated analysis of anatomic joint characteristics, which may be of clinical relevance when planning a potential alignment corrective osteotomy in the treatment of OCD to improve the survival of a concomitant cartilage therapy.

The results of this study underline findings of previous radiologic investigations demonstrating the importance of mechanical LLA in the development of OCD. Similarly, previous studies showed that the mFA-mTA ranges on average between −1.8°± 2.8° and −3°± 0.3° of varus for an MFC-OCD lesion and between 1°± 0.6° and 3°± 3.3° of valgus for an LFC-OCD lesion in comparable mixed adolescent and adult patient populations.^7,18^ Accordingly, the JLCA, a parameter indicative of compartment overload,^29^ could be an independent predictor of OCD lesion location.

While these observations may seem intuitive, malalignment of the mechanical axis may not be the sole factor predictive of the location of the OCD. In accordance with the present study, Brown et al^7^ highlighted, in their radiologic investigation, that a varus and valgus malalignment was associated with a location of the MFC and LFC in only 68% and 67% of the cases, respectively. Gonzalez-Herranz et al^15^ observed a coincidence of the mechanical axis and the location of the OCD lesion in only 31% of cases in their pediatric population. Interestingly, there was no correlation between lesion size and mechanical axis deviation.^7,15^ Investigating additional parameters, Brown et al^7^ demonstrated that the mLDFA, indicative of a femoral-based deformity, was associated with the LFC-OCD; however, the mMPTA did not significantly differ between patients affected by LFC-OCD and MFC-OCD. Accordingly, in the present study, an association between the mMPTA and OCD lesion location could not be detected.

While the mMPTA indicates a tibial-based deformity, it is not the same as the KJLO, and the utility of quantifying knee joint line orientation based on the mMPTA is limited.^23^ When considering the KJLO, biomechanical research has shown that a lateral downsloping of the tibial knee joint line creates a lateral momentum for the femur, resulting in mediolateral tibiofemoral subluxation and pressure distribution changes.^32^ More specifically, the areas of peak pressure have moved toward the downhill meniscus and the uphill tibial spine.^32^ This mechanical engagement of the tibial spine, which serves as a restraint to the coronal translation of the femur, may support the tibial spine impingement theory.^9^ While not reaching statistical significance in the present study—potentially because of a type 2 error—there was a tendency toward a more medially oriented downsloping of the tibial plateau in LFC-OCD (see Table 1), addressing a relevant gap in the literature. In contrast, a lower degree of medial coronal downsloping was observed in patients with MFC-OCD, with an absolute difference of 1.9° between patients with MFC-OCD and LCD-OCD. Although there was no absolute lateral downsloping of the tibia on bipedal weightbearing hip-knee-ankle radiographs in the MFC-OCD cohort, there may exist a tendency toward lateral downsloping during single-leg weightbearing in the stance phase of gait, accounting for the knee adduction moment during this phase, in which the joint line is leveled in a physiological anatomic situation.^2^

Wechter et al^33^ assessed the coronal tibial slope of the respective femorotibial compartment with a proprietary measurement technique in which they used the distance between the tibial spine and the medial/lateral tibial plateau. Interestingly, they found a significantly greater medial downsloping of the medial plateau in patients with MFC-OCD,^33^ which may indicate tibial prominence rather than a true coronal tibial downsloping. However, with a mean difference of 0.9° compared with the control group, the effect size of this measurement was minimal, potentially indicating a nonexclusive role in the etiopathogenesis of OCD.

In our attempt to identify more powerful mechanical predictors of OCD lesion location in the present study, we found various parameters quantifying tibiofemoral subluxation to be highly predictive of the condyle affected with OCD lesion, addressing a further gap in the literature. An analysis of subluxation of the tibia relative to the femur, indicated both by anatomic axes (C-subluxation, rotation angle) as well as mechanical axes (TFJCD), provided evidence that this subluxation reduces the distance between the medial tibial spine and the lateral border of the MFC in MFC-OCD and vice versa in LFC-OCD, which may increase the risk for tibial spine impingement. More specifically, TFJCD was observed to be an independent predictor of OCD location (OR, 0.443 [95% CI, 0.219-0.896]), indicating substantial negative predictive capability for an MFC-OCD lesion. Further, medial tibiofemoral subluxation of the tibia relative to the femur—as quantified by the C-subluxation—was significantly correlated with MFC-OCD lesion size. As the mechanical alignment is not directly associated with lesion size,^7,15^ the cutoff values in subluxation parameters—such as C- and L-subluxation, TFJCD, and rotation angle—may serve as additional parameters in determining which condyle may be at risk for developing OCD.

**Figure 3.**
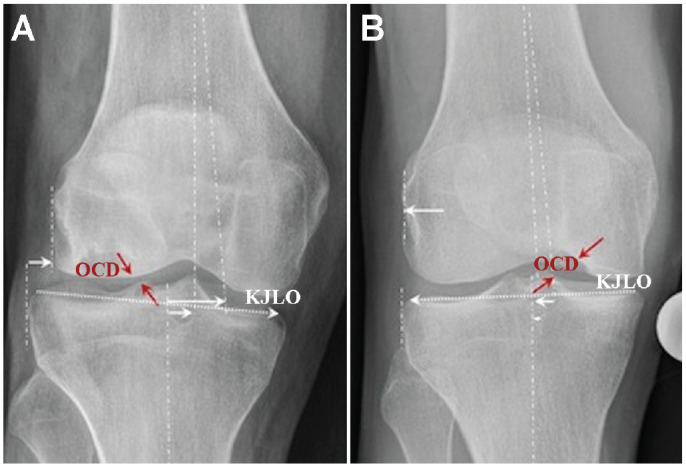
(A) A patient with a lateral femoral condyle OCD lesion in which a lateral tibial subluxation relative to the femur and a medial downsloping of the tibial KJLO (dotted line) may be responsible for an impingement between the lateral femoral condyle and the lateral tibial spine. (B) A patient with a medial femoral condyle OCD lesion in which a medial tibial subluxation relative to the femur and a lateral downsloping of the tibial KJLO (dotted line) may be responsible for an impingement between the medial femoral condyle and the medial tibial spin. KJLO, knee joint line obliquity; OCD, osteochondritis dissecans.

Acknowledging a potentially multifactorial mechanical etiopathogenesis for OCD, varus malalignment,^7,15,18^ as well as medial tibial subluxation relative to the femur and potentially lateral downsloping of the tibial KJLO (Figure 3A), may all have reinforcing effects on the anatomic propensity of developing MFC-OCD. In contrast, patients with valgus malalignment,^7,15,18^ lateral tibial subluxation, and, potentially, medial downsloping of the tibial KJLO (Figure 3B) may be at increased risk of developing LFC-OCD. Collectively, these LAA-related factors may amplify to the effect of a prominent tibial spine,^9^ a narrow intercondylar notch width,^11^ and external tibial torsion^6^ that have been associated with developing OCD.

Of clinical relevance, this study provides a differentiated analysis of anatomic joint characteristics, which may be of clinical relevance when planning a potential alignment corrective osteotomy in the treatment of OCD to improve the survival of a concomitant cartilage therapy.^16,19,26^

### Limitations

The findings of this study must be interpreted within the context of this study’s limitations. First, measurements of patients affected by OCD could not be compared against a healthy control group because of the strict radiation protection protocols at the authors institution in this relatively young patient group. As this is a known issue in radiological studies investigating alignment on OCD,^7,15^ a comparison between patients with MFC-OCD and LFC-OCD was performed, according to previous studies.^7,15^ Second, in accordance with the institutional radiation protection protocols, undergoing a standing long-leg radiograph at our institution reflects the intention to treat, given the relatively high radiation exposure for this young patient cohort. This may introduce a selection bias regarding the severity of the OCD as well as obvious discrepancies between the location of the OCD and the type of LLA deformity. Third, while the sample size was comparable to previous studies,^7^ there might be a risk for a statistical type 2 error for certain measurement parameters given the limited patient population because of the difficulty of acquiring long-leg radiographs in adolescent patients according to institutional radiation protection guidelines. Finally, while strict imaging protocols were used to ensure optimal radiographic quality, measurement accuracy may have been limited because of variances in lower extremity position during image acquisition.

## Conclusion

In this cohort, mechanical LLA was significantly associated with the location of symptomatic OCD. In particular, the position of the tibia relative to the femur was significantly different between patients with MFC-OCD and LFC-OCD. In patients with MFC-OCD, the tibia subluxated medially, resulting in a change of joint geometry by approximating the medial tibial eminence toward the MFC. Interestingly, the extent of subluxation was correlated with the size of the OCD. This finding potentially supports the tibial spine impingement theory, as it suggests an articular overload of the femoral condyle at the tibial eminence and may be a parameter of particular clinical relevance when evaluating mechanical alignment correction in OCD treatment.

## Appendix

**Appendix Table A1:** Interrater and intrarater Reliability of the Radiographic Measurements^*a*^

Measurement	ICC^*b*^	
	Interrater	Intrarater
mLPFA	0.971	0.905
mLDFA	0.972	0.980
mMPTA	0.985	0.951
mLDTA	0.983	0.941
JLCA	0.881	0.912
AMA	0.943	0.882
mFA-mTA	0.992	0.998
Rotation angle	0.974	0.844
KJLO	0.897	0.864
AJLO	0.993	0.992
C-subluxation	0.898	0.896
M-subluxation	0.816	0.989
L-subluxation	0.953	0.913
Tibial plateau width	0.989	0.980
TFJCD	0.915	0.929

a AMA, anatomic mechanical femur angle; AJLO, ankle joint line obliquity; C-subluxation, central subluxation; ICC, intraclass correlation coefficient; JLCA, joint line convergence angle; KJLO, knee joint line obliquity; L-subluxation, lateral subluxation; M-subluxation, medial subluxation; mFA-mTA, mechanical femorotibial angle; mLDFA, mechanical lateral distal femur angle; mLDTA, mechanical lateral distal talus angle; mLPFA, mechanical lateral proximal femur angle; mMPTA, mechanical medial proximal tibia angle; TFJCD, tibiofemoral joint line center distance.

b ICC values were graded as follows: <0.4, poor reliability; 0.4-0.75, moderate reliability; and >0.75, excellent reliability.
